# Use of Electronic Noses for Diagnosis of Digestive and Respiratory Diseases through the Breath

**DOI:** 10.3390/bios9010035

**Published:** 2019-02-28

**Authors:** Carlos Sánchez, J. Pedro Santos, Jesús Lozano

**Affiliations:** 1 Institute of Physics Technology and Information (CSIC), 28006 Madrid, Spain; carlos@updevices.com (C.S.); jp.santos@csic.es (J.P.S.); 2 Up Devices and Technologies, 28021 Madrid, Spain; 3 Industrial Engineering School, University of Extremadura, 06006 Badajoz, Spain

**Keywords:** electronic nose, gas sensors, biomarkers, diseases, digestive system, respiratory system, volatile organic compounds, breath

## Abstract

The increased occurrence of chronic diseases related to lifestyle or environmental conditions may have a detrimental effect on long-term health if not diagnosed and controlled in time. For this reason, it is important to develop new noninvasive early diagnosis equipment that allows improvement of the current diagnostic methods. This, in turn, has led to an exponential development of technology applied to the medical sector, such as the electronic nose. In addition, the appearance of this type of technology has allowed the possibility of studying diseases from another point of view, such as through breath analysis. This paper presents a bibliographic review of past and recent studies, selecting those investigations in which a patient population was studied with electronic nose technology, in order to identify potential applications of this technology in the detection of respiratory and digestive diseases through the analysis of volatile organic compounds present in the breath.

## 1. Introduction

The relationship between aromas present in the breath and disease has been known by doctors for a several hundred years. The use of aromas to identify diseases dates back to the fourth century, when the doctor, based on experience, could determine what disease a patient was suffering. For example, a fruity aroma in the breath was identified as a sign of ketoacidosis associated with diabetes, and the smell of ammonia can be indicative of kidney failure. This method was not accurate, as it was necessary for the disease to be in an advanced stage to be detected by human olfaction. Based on this, electronic systems have been developed to diagnose diseases through the breath and to provide information about the state of the human body [1]. Human breath has a considerable amount of volatile organic compounds (VOCs) that are a product of metabolic activity. These VOCs can differ according to genetic or environmental factors such as age, weight, sex, lifestyle, or eating habits, and can influence the chemical composition of a person’s breath, depending on the amount and concentrations of these compounds. Diseases can also cause an alteration of VOCs in the breath [2].

Currently, there has been an exponential development of technology in medical applications, in both the prevention and diagnosis or treatment of disorders. The growth in areas such as chemical sensors, microelectronic designs, material sciences, and artificial intelligence is contributing to the development of medical technology, and to improved health and increased life expectancy of the population. According to the World Health Organization (WHO), the average life expectancy in the world increased by five years in the period 2000–2016 [3]. The problem is that quality of life has not improved as much as life expectancy. The average increase in life years does not mean that this corresponds to a good state of health. People can suffer diseases and problems that cause a loss of quality of life without endangering their lives. On the other hand, health inequalities persist among different countries and people at different income levels [4]. 

One of the biggest challenges of modern medicine is to develop equipment that allows early diagnosis in a noninvasive way. This could avoid disease exacerbation, permitting control of the evolution of chronic patients in the medium and long term and maintaining their quality of life. It also would contribute to a reduction in hospital costs, as it could reduce the number of hospital/health center visits and the amount of medicine used. The principal limitation of the early detection of disease is that it is impossible to analyze healthy persons continuously, because this would be economically infeasible. In addition, the health system does not have the resources or the medical staff for this purpose. For this reason, the devices should be cheap and portable, so they can be available to more hospitals and health centers. On the other hand, this would open the possibility for patients to have devices at home to perform regular analysis.

These arguments and necessities have motivated the development of new technologies such as the electronic nose. The first electronic nose was developed in 1964 by Wilkens et al. [5], but it was not until 1982 when the electronic nose as a system using chemical sensors to classify odors was described by Persaud et al. [6]. In the 1980s, there was an evolution of this technology, which led to the creation of a technological sector that tried to imitate the olfactory systems of mammals [7]. Since then, the development of electronic noses has gone hand-in-hand with the technology being used in various sectors and different applications [8]. One is the medical sector, particularly the diagnosis and control of respiratory and digestive diseases. This technology could offer the possibility of diagnosing or evaluating disease states in a noninvasive and quick way with low-cost instruments. 

## 2. The Olfactory Organ and Electronic Nose 

Living organisms receive information about the surrounding environment through different sensory organs. Nowadays, the scientific community is focusing on the development and generation of systems and devices that can mimic the sensory organs: first, to replace the function of one of these organs in case of malfunction due to atrophy, pathology, or accident; and second, to be used in a wide variety of industrial applications in fields such as medicine, agriculture, food, environment, etc. Given that a deficiency in the ability to smell does not limit a person’s normal life, except for the detection of gas leaks or fires, this technology becomes increasingly important in other applications [9,10].

**Figure 1:**
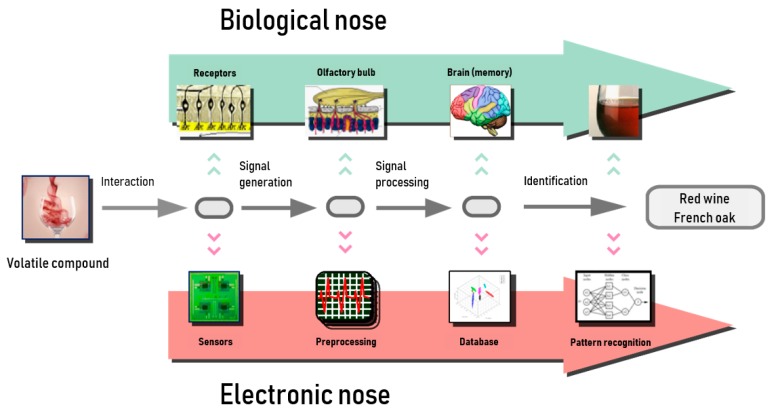
Schematics of electronic nose and biological olfactory system [13].

The electronic nose is capable of detecting, discriminating, and identifying different types of chemicals present in the headspace of a sample as VOCs. The device’s response to smell is produced by the interaction between volatile compounds and sensors, whereas this function is done in the biological nose by the olfactory epithelium, which works as a transductor, as it generates electrical signals from chemical stimuli. These signals are preprocessed in the olfactory bulb, then transmitted to the brain, where they are stored. Finally, the data are used to identify odors in the learning stage. Analogously, in an electronic nose, an analogical–digital conversion is produced to preprocess the signals, with a microcontroller employed for this purpose. In this case, the data are stored in the database of a pattern recognition machine to identify the aromas that are learned. Figure 1 shows a comparison between the electronic nose and biological nose [10,11]. 

Due to the wide variety of applications that have been generated recently in several sectors, sensors based on different detection principles have been developed to satisfy the needs that have arisen. Sensors undergo a physical or chemical change by interacting at the molecular level when exposed to a gas. This process is reversible and allows the sensors to be used again in other tests [8]. A wide variety of sensors have been developed: metal oxide semiconductor (MOS), conducting polymer (CP), chemocapacitor (CAP), electrochemical (EC), metal oxide semiconductor field effect (MOSFET), surface acoustic wave (SAW), quartz crystal microbalance (QCM), bulk acoustic wave (BAW), fluorescence (FL), optical fiber live cell (OF-LC), catalytic field effect (CFET), calorimetric or catalytic bead (CB), carbon black composite (CBC), micro-electromechanical system (MEMS), photoionization detector (PID), and amperometry gas sensor (AGS) [12].

This offers a wide range of possibilities, so it is possible to use one type of sensor or another according to the application [8,12]. For example, sensors with good sensitivity are MOS, MOSFET, and AGS. SAW, FL, and OF-LC combine sensibility with specificity. However, the principal disadvantages of the gas sensor are slow recovery (MOS), drift in the response (MOS, CP, SAW), low noise immunity (PID), and lack of reproducibility between sensors of different sets (CP, MOSFET, QCM, SAW) and in the response of the same sensor in the long term. Because of this, it is normal to use a matrix of sensors of different types to avoid the disadvantages presented by each one separately and maximize their advantages [12,14]. 

## 3. Biomarkers and Diseases

In 1899, Thomson, who was interested in measuring the mass/charge ratio of an electron, created the first instrument similar to a mass spectrometer (MC), improving on the work previously done by Wien. In 1941, Martin and Synge published a paper describing the liquid–liquid chromatographic partition. However, it was not until 1952 when chromatography took on its gas–solid version (James and Martin). In later years, equipment was developed that used both techniques, which eliminated the disadvantages that each one presented separately [15,16]. One of the main problems encountered in the analysis of VOCs was the impossibility of capturing these compounds, as they are very volatile. This inconvenience was solved with the emergence of solid-phase microextraction (SPME) [17]. Since then, new techniques have been developed, such as gas chromatography–mass spectrometry (GC-MS), proton transfer reaction–mass spectrometry (PTR-MS), selected ion flow tube–mass spectrometry (SIFT-MS), field asymmetric ion mobility spectrometry (FAIMS), time-of-flight mass spectrometry (TOF-MS), ion mobility spectrometry (IMS), liquid chromatography–mass spectrometry (LC-MS), and high-performance liquid chromatography (HPLC). 

With these techniques, it is possible to extract information about the respiratory or digestive system through the breath. However, the existence of VOCs in respiration does not necessarily imply that these volatile molecules are produced by the human body; rather, they can be produced in an exogenous process. For example, acetonitrile is commonly found in the breath of smokers, occurring exogenously. Exposure to a dangerous atmosphere, contaminants, or even certain medications can generate new compounds and also alter the concentration of other endogenous compounds [18].

Table 1 shows descriptions of different processes associated with oxidative stress and airway inflammation and the influence of these processes on the composition of VOCs in the breath. Endogenous compounds are produced by respiratory or digestive system cells, which metabolize the molecules present in the inspired air and generate others. When the cells function abnormally, they can alter the composition of VOCs in the breath. For example, oxidative stress is an imbalance of the normal state of the human body caused by the production of free radicals, which alters the composition of the compounds present in the breath as H2O2, CO, or 8-isoprostane [19,20]. Another example is the inflammation of airways, where the immune response produces an increase of various biomarkers in the breath [19,21].

Microorganisms that may be present in the digestive or respiratory tract, such as viruses, bacteria, or fungi, can also directly or indirectly alter the composition and concentration of VOCs in the breath. These cannot modify the concentration by themselves; otherwise, these microorganisms can affect the normal functioning of the cells of the body, causing the same effect. Therefore, the alteration of volatile compounds can have an exogenous or endogenous cause, which can be produced by a malfunction of the cells or by external biological agents [18]. 

### 3.1. Endogenous Biomarkers

The following section describes the pathologies detected by different chemical techniques of analysis that are mentioned in the bibliography consulted. Asthma and chronic obstructive pulmonary disease (COPD) are chronic diseases that cause difficulty breathing due to inflammation of the airways, which become rigid [24,25]. Inflammation of the epithelial cells causes increased concentrations of NO, pentane, isoprene, and ethane in the expired air in asthma (Lärstad et al. [26]) and ethane in the expired air in COPD (Paredi et al. [27]) (Table 2). This occurs with other diseases described in the bibliography, such as acute respiratory distress syndrome (ARDS), cystic fibrosis (CF), and lung cancer. ARDS is a pathology that prevents enough oxygen from reaching the lungs and the bloodstream [28]. CF is a genetic and chronic disorder that affects organs such as the pancreas, liver, kidneys, and intestine, causes difficulty breathing, and generates dense mucus in the airways [29]. A study by Antuni et al. [30] used NO and CO as biomarkers for the discrimination of this disease. In the case of NO, the concentration of this biomarker decreases in patients with CF in comparison to healthy individuals. In the case of CO, the concentration increases. 

Finally, in lung cancer, the process of cell division is disrupted by the creation of new cells in an uncontrolled way and when they are not necessary, as well as allowing old or damaged cells to survive [31]. Bajtarevic et al. [32] employed isoprene, acetone, methanol, and benzene as biomarkers of lung cancer. The concentrations of these compounds decreased in patients with this disease in comparison with healthy persons. Table 2 shows chemical analysis techniques and biomarkers used to detect different diseases.

As already mentioned, diseases of the digestive system, such as diabetes mellitus, liver fibrosis, and hypolactasia, can alter the concentration of VOCs in the same way as respiratory diseases. Diabetes is a disease in which the body does not produce insulin or does not use it correctly [42]. Liver fibrosis is a disorder that causes decreased blood supply through the liver and produces the accumulation of scar tissue [43]. Das et al. [37] and Kearney et al. [39] used acetone as the major biomarker to detect both pathologies. The concentration of this compound increased in patients with these disorders in comparison with healthy persons. Lastly, hypolactasia is a deficiency of lactase (the enzyme that metabolizes lactose) in the intestinal mucosa, which causes this molecule to not be metabolized [44]. For the detection of this disorder, Alkhouri et al. [41] used hydrogen as a biomarker.

### 3.2. Exogenous Biomarkers

In this section, diseases generated by external pathogens, such as pneumonia and pulmonary tuberculosis, which produce exogenous volatile organic compounds, are described. Pneumonia is a lung infection that can be caused by many pathogenic agents, such as bacteria, viruses, or fungi [45], while tuberculosis is a bacterial infection (Mycobacterium tuberculosis) that mainly attacks the lungs or other parts of the organism [46]. However, it should be noted that there are diseases (e.g., cystic fibrosis and COPD) that predispose the patient to infections such as pneumonia [29]. Therefore, in this pathology it is common to find endogenous and exogenous VOCs in the breath. In order to determine whether a person is ill, it is necessary to know the aromatic profile of a healthy person. The concentrations of existing major compounds in the breath of healthy persons can be seen in Table 3.

## 4. Traditional Methods of Diagnosis

Traditionally, chemical analysis techniques have not been used to diagnose respiratory or digestive diseases, as these are very expensive. Breath analysis may mitigate some of the disadvantages of conventional diagnostic tests. Additionally, it could complement conventional methods as a screening tool. The main disadvantage of traditional diagnostic tests is that there is usually a long wait to conduct the test. In addition, some are invasive tests, which may require irradiation of the patient or a surgical procedure. In other cases, the test result is not obtained immediately, as in the case of cultures.

Both invasive and noninvasive diagnostic techniques are currently used. For example, spirometry and fractional exhaled nitric oxide (FeNO) are noninvasive techniques used to determine the patient’s lung capacity, mainly used to diagnose diseases such as asthma and COPD. The sweat test and sputum cultures are also employed as noninvasive techniques for the diagnosis of cystic fibrosis (described by Gibson and Cooke [48] in 1959) and respiratory infections, respectively. On the other hand, diagnostic tests used to detect other diseases described in the bibliography are lung biopsy (lung cancer and lung sarcoidosis), chest x-ray (CXR) (ARDS and lung tuberculosis), endoscopy (lung cancer), and computed tomography (CT) (lung sarcoidosis and lung cancer). 

## 5. Recent Developments in Electronic Noses for the Diagnosis of Respiratory Diseases

One of the major objectives in medicine today is to develop equipment and techniques to achieve early diagnosis of diseases. For this, it is necessary to develop devices that are portable, economical, and, above all, noninvasive, in order to reduce the risk to patients. In addition, this would contribute to decentralizing medical resources and facilitate access to these devices, reducing waiting lists. Until now, noninvasive tests that have been performed were based only on the chemical analysis of body fluids (feces, urine, and blood), but the possibility of analyzing the volatile compounds generated by the samples (as well as exhaled air) has not been explored.

Traditionally, chemical analysis techniques such as GC-MS and, to a lesser extent, the techniques described in Section 3 have been used to detect VOCs and the chemical composition of gaseous samples. With the evolution of gas sensors and the electronic nose in recent decades, they are beginning to be used as diagnostic methods. It is true that currently gas sensors are not as selective as chemical analysis equipment, but the electronic nose allows for much smaller and compact equipment, which offers a lot of possibilities. 

This paper includes a summary of the specialized bibliography on the detection of respiratory and digestive diseases through the breath using an electronic nose (Table 4). It should be noted that the scientific research carried out in recent years is included here. Therefore, only the electronic noses used in such studies are described, with the intention that this paper may help to determine the current state of research in the field and be a starting point for future studies. In the bibliography, it is possible to find some authors who have used commercial electronic noses and others who have developed their own equipment adapted to specific applications. It should be noted that the diagnostic tests employed are used to corroborate the results of the studies, but they do not have to be standard tests. Most of the authors described in this paper used a commercial electronic nose, the Cyranose 320, which has a matrix of 32 sensors of carbon black polymer. Only a few used a self-developed electronic nose optimized for a specific application.

Asthma is one of the respiratory diseases that affects the largest number of people in the world, based on more papers being found on this disease. The population studied by Dragonieri et al. [49,50] was 40 adult subjects, and they employed the Cyranose 320. In data processing, they used principal component analysis (PCA) as an alternative diagnostic test to corroborate the results obtained by using spirometry and FeNO. The other authors studying asthma used similar population sizes, and also used the Cyranose 320. They used spirometry or FeNO as a diagnostic test. While not all authors used the same techniques in the processing phase, they employed PCA and other techniques such as receiver operating characteristic (ROC) curve and neural network algorithms (ANNs). On the other hand, Brinkman et al. [51] studied a smaller population of subjects and performed the measurements using three electronic noses, two commercial (Cyranose 320, carbon black polymer sensors) and Tor Vergata Electronic Nose (QCM sensors; developed by the Sensors Group at the University of Rome Tor Vergata, along with the Departments of Electronic Engineering and Chemical Science and Technology), and one home-developed incorporating metal oxide semiconductor sensors.

In the case of COPD, a variety of scientific articles were found that used the same two commercial electronic noses as in the previous case: Cyranose 320 and Tor Vergata Electronic Nose. Dymerski et al. [52] used an electronic nose formed by commercial sensors from Figaro. In this case, the samples were generated in the laboratory, unlike in the other studies. To distinguish between patients and healthy individuals, Paredi et al. [35] employed three biomarkers: ethanol, CO, and NO, the concentrations of which in the exhaled air of patients with COPD increased compared to the healthy people. The literature found for lung cancer varies in terms of the type of cancer described; each author focused on the detection of one, studying populations of between 50 and 100 adult patients, using the commercial electronic noses that were used by other authors.

In the detection of pulmonary tuberculosis, a wider variety of authors were found to use different electronic noses with different types of sensors: commercial ones such as Cyranose 320, Aeonose, and Bloodhound BH1114, and home-developed equipment, such as Zelota et al. [53] using QCM, Bruins et al. [54] using metal oxide semiconductor sensors, and Fend et al. [55] using conducting polymer sensors. 

For the rest of the pathologies shown in the table, fewer bibliographical references were found. Saasa et al. [56] discriminated between samples of patients with diabetes mellitus by detecting acetone. On the other hand, Schnabel et al. [57] stand out, discriminating between patients with pneumonia and healthy people. They studied a remarkable sample of 125 adult patients using a commercial electronic nose called DiagNose, which uses metal oxide sensors.

## 6. Future and Challenges

Commercial electronic noses, used by most researchers, are designed for general use and are not optimized to detect the biomarkers of the diseases of interest. For medical applications, it is necessary to design specific sensors and optimized electronic noses, since it is an extremely complex sector, and it is a critical factor for the diagnosis and control of pathologies to be effective. In the medical sector, it is necessary to develop equipment that allows early diagnosis of diseases, enables simple and effective control, and is noninvasive. 

On the one hand, this would allow health centers and small hospitals to have access to this type of equipment for the diagnosis and control of diseases (due to its lower cost) and thus minimize waiting lists. On the other hand, this would provide an opportunity for patients to have devices at home. In addition, in conjunction with the evolution of mobile technologies and Internet communication, it would allow patients to diagnose and control diseases from home by themselves or with remote medical supervision. It could even permit these types of sensors to be connected to phones either by incorporating them or through a gadget. The tendency of medicine is to advance along this line so that analyses and explorations can be performed at a distance. It is hospitals and health insurers themselves that could provide these types of devices to patients. In this way, it would be possible to achieve a unified protocol of measurement from the breath that is simple for patients to perform.

It is a promising technology for the diagnosis of respiratory and digestive diseases in a noninvasive way, since it allows the development of portable and cost-effective equipment. This is a key factor for this technology to succeed. However, current sensors still present complications that limit their operation. Therefore, one of the challenges from a technological point of view is to improve the selectivity of these sensors and their sensitivity to lower concentrations. To this end, it is important to use and develop new nanomaterials that can significantly improve selectivity and reduce the size and consumption, essential issues for miniaturization. It is therefore perhaps more important to focus efforts on developing technology that would enable these devices to be used in this application, and then study which is the ideal methodology to take samples in patients that is easy for a person without medical education to do. 

## 7. Conclusions

Electronic nose technology has evolved remarkably in the last decade. Many researchers have focused their efforts on developing this type of equipment in a multitude of sectors, such as food, medicine, environment, and detection of hazardous materials.

This study presents a bibliographic review of research done on the use of the electronic nose for the detection of respiratory and digestive diseases through the breath. This technology bases its design and operation on the human sensory organ. The e-nose technology permits the study of diseases from a new approach, since until now analytical tests have focused exclusively on blood, urine, or feces samples, which allowed additional information to be obtained and studied. Although this technology is still in development and currently has some limitations, it has several advantages over other diagnostic methods. The ability to take measurements through the breath makes it possible to conduct analyses noninvasively, eliminating risks to the patient.

In addition, it would allow the production of portable equipment at a lower price than other equipment available in the market, giving health centers and hospitals greater access to this equipment. With the evolution in recent years and current development (improvement and miniaturization of gas sensors), the electronic nose is presented as a promising technology that should contribute to improving the quality of life of patients with chronic pathologies as well as early diagnosis of different diseases, contributing to the reduction of direct and indirect costs of the health system. 

The purpose of this paper is to demonstrate the viability of this technology through a large number of studies done in this regard. From now on, it will be necessary to improve and solve the problems presented by the e-nose, fine-tuning this technology for the different applications in which it is intended to be used. This will require a great effort on the part of the research community, but once these problems have been solved, this technology should result in a great advance in the control and diagnosis of diseases, and satisfy many other needs that currently exist in the medical sector.

## Figures and Tables

**Table 1:** Processes involved in different pathologies.

Process	Description	Biomarker	References
Marker of oxidative stress	Inflammation process in lung cells; eosinophils, neutrophils, and macrophages produce reactive oxygen species	H_2_O_2_	[19,22,23]
	Increase of free radicals, which react to cell membrane phospholipid, generating 8-isoprostane	8-isoprostane	[19,20]
	Oxidation of cell membrane phospholipids produces a chain reaction, the targets of which are polyunsaturated fatty acids, resulting in the formation of unstable lipid hydroperoxides and secondary carbonyl compounds, such as aldehydic products	Malondialdehyde	[19]
	CO, a marker of oxidative stress, is produced by the stress protein hemoglobin oxygenase	CO	[21]
Inflammation of airways	Immune response against infection produces an inflammation process in cells, which generate more NO than in a healthy person	Alveolar NO	[19]
	Cytokines * and chemokines are involved in many aspects of the disease process in chronic obstructive pulmonary disease (COPD), including recruitment of neutrophils, macrophages, T cells, and B cells	Cytokines * and chemokines	[19]
	Leukotrienes are muscle constrictors, such as in lung muscle	Leukotriene B4 and prostaglandins	[19]
	CO is a marker of inflammation	CO	[21]

* Cytokines are agents responsible for cellular communication.

**Table 2:** Concentrations of biomarkers used in the detection of different diseases. NS, not stated; ppb, parts per billion; ppmv, parts per million by volume.

Disease	Study	Biomarker	Concentration		References
Asthma	Lärstad (2007)	Ethane	NS		[26]
		NO	19 ± 2 ppb (healthy subject); 30 ± 6.1 ppb (asthma patient)		
		Pentane	NS		
		Isoprene	113 ppb		
	Olopade (1997)	Pentane	Acute asthma: 8.4 ± 2.9 nmol/L		[33]
		Pentane	Stable asthma: 3.6 ± 0.4 nmol/L		
	Paredi (2000)	Ethane	Ethane: asthma not treated with steroids: 2.06 ± 0.30 ppb; asthma treated with steroids: 0.79 ± 0.1 ppb); healthy volunteers: 0.88 ± 0.09 ppb		[27]
			NO: asthma not treated with steroids: 14.7 ± 1.7 ppb; asthma treated with steroids: 8.6 ± 0.5 ppb		
	Dweik (2011)	NO	Low asthma patients: <25 ppb in adults; >20 ppb in children Intermediate asthma patients: 25–50 ppb in adults; 20–35 ppb in children High asthma patients: >50 ppb in adults; >35 ppb in children Persistently high asthma patients: >50 ppb in adults; 35 ppb in children		[34]
COPD	Paredi (2000)	Ethane	2.77 ± 0.25		[35]
Cystic fibrosis	Barker (2006)	Pentane	0.36 (0.24–0.48) ppb		[36]
		Dimethyl Sulfide	3.89 (2.24–5.54) ppb		
	Antuni (2000)	NO	Healthy volunteers: 7.3 (0.24) ppb; stable cystic fibrosis patients: 5.7 (00.29) ppb; unstable cystic fibrosis patients: 6.1 (0.72) ppb		[30]
		CO	Healthy volunteers: 2.0 (0.1) ppm; stable cystic fibrosis patients: 2.7 (0.22) ppm; unstable cystic fibrosis patients: 4.8 (0.3) ppb		
Lung cancer	Bajtarevic (2009)	Isoprene	Median concentration: healthy volunteers: 105.2 ppb; cancer patients: 81.5 ppb		[32]
		Acetone	Median concentration: healthy volunteers: 627.5 ppb; cancer patients: 458.7 ppb		
		Methanol	Median concentration: healthy volunteers: 142.0 ppb; cancer patients: 118.5 ppb		
		Benzene	Median concentration: healthy volunteers: 627.5 ppb; cancer patients: 458.7 ppb		
Diabetes mellitus	Das (2016)	Acetone	Type 1	0.044–2.744 ppm (healthy volunteers); 2.2–21 ppm (diabetes patients)	[37]
			Type 2	0.044–2.744 ppm (healthy volunteers); 1.76–9.4 ppm (diabetes patients)	
	Spanel (2011)	Acetone	Type 2	<800 ppb (healthy volunteers); >1760 ppb (diabetes patients)	[38]
Helicobacter pylori	Kearney (2002)	Dioxide carbon and ammonia.	NS		[39]
Hypolactasia	Metz (1975)	Hydrogen	Control: 0–3 ppmv; patients: 48–168 ppmv		[40]
Liver fibrosis	Alkhouri (2015)	Acetone	Lower fibrosis group: 117.8 ppb; advanced fibrosis group: 224.2 ppb		[41]
		Benzene	Lower fibrosis group: 1.9 ppb; advanced fibrosis group: 8 ppb		
		Carbon Disulfide	Lower fibrosis group: 1.6 ppb; advanced fibrosis group: 3.2 ppb		
		Isoprene	Lower fibrosis group: 13.5 ppb; advanced fibrosis group: 40.4 ppb		
		Pentane	Lower fibrosis group: 12.3 ppb; advanced fibrosis group: 19.5 ppb		
		Ethane	Lower fibrosis group: 63.0 ppb; advanced fibrosis group: 75.6 ppb		

**Table 3:** Major volatile organic compounds present in the breath of healthy individuals [47].

Compound	Concentration
Water vapor	5–6.3%
Nitrogen	78.04%
Oxygen	16%
Carbon dioxide	4–5%
Hydrogen	5%
Argon	NS
CO	0–6 ppm
Ammonia	0.5–2 ppm
Acetone, methanol, ethanol	0.9%; <1 ppm
Hydrogen sulfide	0–1.3 ppm
NO	10–50 ppb
Carbonyl sulfide	0–10 ppb
Ethane	0–10 ppb
Pentane	0–10 ppb
Methane	2–10 ppm

**Table 4:** Biomedical applications developed using commercial and experimental electronic noses.

Application	Author	Population Characteristics	Sensor Technology	Number of Sensors	Data Processing Algorithm	Diagnosis	Other Techniques	References
Asthma	Dragonieri (2007)	40 adult subjects, nonsmokers, aged 18–75, without any other acute or chronic disease besides asthma (mixed group) Group 1: 10 patients, 25.1 ± 5.9 years, intermittent-mild asthma Group 2: 10 patients, 26.8 ± 6.4 years (control group) Group 3: 10 patients, 49.5 ± 12.0 years, moderate-severe persistent asthma Group 4: 10 patients, 57.3 ± 7.1 years (control group)	Polymer nanocomposite sensor	32	PCA	Spirometry, FeNO	GC-MS	[49]
	Montuschi (2010)	52 adult subjects, nonsmokers (mixed group) Group 1: 27 asthma patients, 39 ± 3 years Group 2: 24 patients, 33 ± 3 years (control group)	QCM gas sensors coated by molecular metalloporphyrin film	8	PCA and FNN	FeNO	GC-MS	[58]
	Santonico (2014)	58 subjects	Carbon black polymer (Cyranose C320)/QCM covered with metalloporphyrin film (Tor Vergata Electronic Nose)/metal oxide semiconductor	32/NS/NS	ROC	FeNO	FAIMS (Owlstone)	[59]
	Brinkman (2017)	28 subjects 23 asthma patients, 25 (21–31) years 5 healthy volunteers, control group	Carbon black polymer (Cyranose C320)/QCM covered with metalloporphyrin film (Tor Vergata Electronic Nose)/metal oxide semiconductor	32/NS/NS	PCA	FeNO and spirometry	FAIMS (Owlstone) and GC-MS	[51]
	Cavaleiro (2018)	60 subjects, aged 6 –18 years (mixed group)	Polymer nanocomposite sensor (Cyranose 320)	32	Clustering	FeNO and spirometry	GC-MS	[60]
Chronic obstructive pulmonary disease	Paredi (2000)	36 subjects (mixed group) Group 1: 12 nonsteroid-treated patients, 60 ± 18 years Group 2: 10 steroid-treated patients, 58 ± 2 years Group 3: 14 healthy subjects, 33 ± 3 years (control group)	NS	NS	NS	NS	NS	[35]
	Capuano (2010)	20 subjects (mixed group) Group 1: 12 COPD patients, ex-smokers, therapy not based on cortisone Group 2: 8 healthy volunteers (control group)	QMC gas sensor with metalloporphyrin films	7	PLS-DA	NS	NS	[61]
	Hattesohl (2011)	33 subjects (mixed group) Group 1: 10 COPD patients Group 2: 23 patients (control group)	Polymer nanocomposite sensor (Cyranose 320)	32	LDA, MD, CVVs, canonical plot,	Spirometry	GC-MS	[62]
	Dymerski (2013)	In vitro experiments	SAW/BAW sensors (TGS 880, TGS 825, TGS 826, TGS 822, TGS 2610, TGS 2602 by Figaro)	6	PCA	NS	NS	[52]
	Bofan (2013)	24 adult subjects, ex-smokers, 68 ± 1.7 years, with smoking history of 39.5 (24.2 –63.3) years without other acute or chronic disease besides COPD or nonatopic COPD and without inhaled or oral corticosteroids (mixed group)	Polymer nanocomposite and inorganic conductor sensor (carbon black) (Cyranose 320)	32	Pattern recognition algorithm	Spirometry and FeNO	GC-MS, NMR spectroscopy, and LC-MS	[63]
Acute respiratory distress syndrome	Bos (2014)	180 subjects (mixed group) Group 1: 58 ARDS patients, 57 (54–78) years Group 2: 11 pneumonia patients, 56 (49–62) years Group 3: 19 cardiogenic pulmonary edema patients, 71 (63–79) years Group 4: 92 healthy volunteers, 64 (50–75) years (control group)	Polymer nanocomposite sensor	32	ROC	CXR	GC-MS	[64]
Pulmonary sarcoidosis	Dragonieri (2013)	31 subjects (mixed group) Group 1: 11 patients, 48.4 ± 9.0 years, untreated pulmonary sarcoidosis Group 2: 20 patients, 49.7 ± 7.9 years, treated pulmonary sarcoidosis Group 3: 25 patients, 39.6 ± 14.1 years (control group)	Polymer nanocomposite sensor (Cyranose 320)	32	PCA, CDA ROC curves	CXR, CT, biopsy	GC-MS, TOF-MS, IMS	[65]
Cystic fibrosis	Paff (2013)	48 subjects (mixed group) Group 1: 25 patients, 11.4 years Positive bacterial cultures: 15/25 patients Pulmonary exacerbation: 9/25 patients Group 2: 23 patients, 9.3 years (control group)	Polymer nanocomposite sensor (Cyranose 320)	32	PCA, ROC curves, and CDA	Spirometry and sputum culture	GC-MS	[66]
Primary ciliary dyskinesia	Paff (2013)	48 subjects (mixed group) Group 1: 25 patients, 10.7 years Positive bacterial cultures: 4/25 patients Pulmonary exacerbation: 8/25 patients Group 2: 23 patients, 9.3 years (control group)	Polymer nanocomposite sensor (Cyranose 320)	32	PCA, ROC curves, and CDA	Spirometry and sputum culture	GC-MS	[66]
Lung cancer	Di Natale (2003)	50 subjects (mixed group) Group 1: 42 patients with various forms of cancer not showing any other disease Group 2: 8 patients without respiratory disease not taking any medication	QCM gas sensors coated with metalloporphyrin molecular film	8	PLS-DA	NS	GC-MS	[67]
	Machado (2005)	59 subjects (mixed group) Group 1: 14 patients, 64 ± 3 years, with untreated bronchogenic carcinoma 13 patients with non-small-cell cancer 1 patient with small-cell cancer Group 2: 45 patients, 38 ± 2 years (control group)	Polymer nanocomposite sensor (Cyranose 320)	32	PCA, SVM, and CDA	CXR and biopsy	GC–MS	[68]
	Dragonieri (2009)	30 subjects (mixed group) Group 1: 10 NSCLC patients, 66.4 ± 9.0 years: 2 current smokers, 7 ex-smokers, 1 never smoked Group 2: 10 COPD patients, 61.4 ± 5.5 years: 6 current smokers, 4 ex-smokers Group 3: 10 healthy volunteers, 58.3 ± 8.1 years, never smoked (control group)	Polymer nanocomposite sensor (Cyranose 320)	32	CDA, CVV, PCA	CT	GC–MS	[69]
	Dragonieri (2012)	39 subjects (mixed group) Group 1: 13 patients, 60.9 ± 12.2 years, with confirmed diagnosis of MPM Group 2: 13 subjects, 67.2 ± 9.8 years, long-term professional exposure to asbestos Group 3: 13 subjects, 52.2 ± 16 years, no asbestos exposure (control group)	Polymer nanocomposite sensor (Cyranose 320)	32	CVA, PCA, and ROC	NS	GC–MS	[70]
	D’Amico (2010)	98 adult subjects, 50–70 years (mixed group) Group 1: 56 patients with primary lung cancer, ex-smokers, not under oncological therapy, at least 6 months from last intervention Group 2: 36 patients with normal lung function, negative history of chest symptoms, nonsmokers, no history of respiratory disease (control group)	QCM gas sensors	8	PLS-DA	Endoscopy	GC-MS	[23,71]
Pneumonia	Hockstein (2005)	25 subjects (mixed group) Group 1: 13 patients with diagnosed pneumonia Group 2: 12 patients without pneumonia	Polymer nanocomposite sensor (Cyranose 320)	32	SMV and PCA	CT	GC–MS	[72]
	Chiu (2015)	In vitro experiment	Polymer–carbon composite with polymers on chemical sensor array	8	CRBM	CXR, blood draw, and sputum culture	GC–MS	[73]
	Schnabel (2015)	125 subjects (mixed group) Group 1: 33 pneumonia patients, 62 (20–82) years, subject to BAL Group 2: 39 pneumonia patients, 57 (23–82) years Group 3: 53 patients, 60 (34–85) (control group)	MOS sensors (DiagNose)	NS	PCA and ROC curves	CT	GC–MS	[57]
Pulmonary tuberculosis	Pavlou (2004)	In vitro experiment	Gas-sensor array (Bloodhound BH114)	14	PCA, optimization of BP-FNN, multivariate techniques,	CXR	NS	[74]
	Fend (2006)	330 patients (mixed group) 188 pulmonary tuberculosis patients: 53.7% HIV patients, 31.4% smokers 142 nonpulmonary tuberculosis patients: 29.6% HIV patients, 9.2% smokers	CP sensor	14	PCA, DFA, and ANN	CXR and sputum culture	GC-MS	[55]
	Bruins (2013)	30 patients (mixed group). Group 1: 15 pulmonary tuberculosis patients, 32 (21–58) years Group 2: 15 healthy volunteers, 30 (18–58) years (control group)	MOS sensor: AS-MLC, AS-MLN, AS-MLK, AS-MLV	12 (4 types of sensors in triplicate)	ANN	CXR and microbiological culture	GC-MS	[54]
	Coronel (2017)	110 subjects (mixed group) Group 1: 47 pulmonary tuberculosis patients, 34.6 years Group 2: 14 COPD or asthma patients, 54.5 years Group 3: 49 patients (control group)	MOS sensors (Aeonose)	NS	ROC curve	CXR	GC-MS	[75]
	Zelota (2017)	71 subjects (mixed group) Group 1: 31 pulmonary tuberculosis and HIV patients, 28.7 ± 7.2 years Group 2: 20 pulmonary tuberculosis patients without HIV, 39 ± 9.3 years Group: 20 healthy volunteers, 33 ± 11 years (control group)	QCM gas sensors coated by metalloporphyrin molecular film	8	PCA	CXR	GC-MS	[53]
	Mohamed (2017)	500 patients (mixed group) Group 1: 260 pulmonary tuberculosis patients, 41.72 ± 16.03 years Group 2: 204 healthy volunteers, 43.38 ± 12.42 years (control group)	MOS sensor	10	PCA and ANN	Physical examination and routine laboratory analyses, including CXR	GC-MS	[76]
Diabetes mellitus	Saasa (2018)	NS	QCL, LAP, and chemoresistive sensors	NS	NS	NS	GC-MS, LC-MS, HPLC, PTR-MS, and SIFT-MS	[56]

## Abbreviations

ANN	Artificial neural network
AS-MLC	Metal oxide semiconductor sensor for detection of carbon monoxide, manufactured by Applied Sensor Technologies
AS-MLK	Metal oxide semiconductor sensor, manufactured by Applied Sensor Technologies
AS-MLN	Metal oxide semiconductor sensor for detection of nitrogen monoxide sensor, manufactured by Applied Sensor Technologies
AS-MLV	Metal oxide semiconductor sensor for detection and reduction of gases such as VOCs and CO, manufactured by Applied Sensor Technologies
BAL	Bronchoalveolar lavage
BP	Backpropagation
CDA	Canonical discriminant analysis
CO	Carbon monoxide
CRBM	Convolutional restricted Boltzmann machine (a type of probabilistic neural network)
CVA	Cross-validated accuracy
CVV	Cross-validation value
DFA	Discriminant function analysis
FNN	Feedforward neural network
GC	Gas chromatography
H_2_O_2_	Hydrogen peroxide
LAP	Light-addressable potentiometric
LTB4	Leukotriene B4
MD	Mahalanobis distance
MDA	Malondialdehyde
MS	Mass spectrometry
NMR	Nuclear magnetic resonance
NO	Nitric oxide
PCA	Principal component analysis
PLS-DA	Partial least squares–discriminant analysis
QCL	Quantum cascade laser
ROC	Receiver operating characteristic
SVM	Support vector machine
